# Effects of Probiotic Intervention on Markers of Inflammation and Health Outcomes in Women of Reproductive Age and Their Children

**DOI:** 10.3389/fnut.2022.889040

**Published:** 2022-06-06

**Authors:** Kah Onn Kwok, Lisa R. Fries, Irma Silva-Zolezzi, Sagar K. Thakkar, Alison Iroz, Carine Blanchard

**Affiliations:** ^1^Food Science and Technology Programme, National University of Singapore, Singapore, Singapore; ^2^Nestlé Research, Singapore, Singapore; ^3^Nestlé Research, Beijing, China; ^4^Nestlé Institute of Health Sciences, Nestlé Research, Société des Produits Nestlé S.A., Lausanne, Switzerland

**Keywords:** probiotics, inflammatory markers, gestational diabetes, polycystic ovarian syndrome (PCOS), gestational diabetes (GDM), atopic dermatitis (AD)

## Abstract

The human intestinal microbiota has been shown to be modulated during inflammatory conditions. Probiotic administration has been shown to affect the immune system and cytokine expression which can affect inflammation and health outcomes. There seems to be an association between the mother's intestinal microbiota and inflammation biomarkers, both of which may contribute to newborn early life immune and metabolic programming and impact short and long-term health outcomes. Probiotic supplementation during pregnancy has been shown to influence metabolic health, immunity, and gastrointestinal health of the mother, and can also have carry-over benefits to infants such as infant allergy risk reduction. Therefore, this review focuses on the evidence of probiotic administration in women of reproductive age, including during pregnancy and its impact on inflammatory markers and on maternal and infant health. We performed a PubMed search for articles published in English in the last 20 years. Immune markers were narrowed to serum and breast milk levels of TNF-α, IL-6 and TGF-β, IgA, and IL-10. Studies that investigated the beneficial effects of interventions in women with gestational diabetes mellitus, polycystic ovarian syndrome, and infant allergy management are summarized. These results show a beneficial or neutral effect on selected health outcomes and that it is safe for woman and their infants. The effect of probiotics on modulation of inflammatory markers was probiotic specific. More research is needed to further our understanding of the mechanisms underlying the effects of probiotics on inflammation and how these effects improve health outcomes.

## Introduction

Globally, women suffer from autoimmune diseases and inflammatory diseases more often than men; level of estrogen, puberty, pregnancy, and menopause are associated with the increased risk (1). During pregnancy, immunological changes can occur at the placental interface to inhibit the rejection of the fetus. Simultaneously, at the mother's mucosal surface, there can be elevated inflammatory responses which can result in autoimmune diseases (2). The mother's intestinal microbiota, body weight and metabolic biomarkers appear to be interlinked and these can contribute to the seeding of a healthy microbiota of the newborn at birth and may affect the infant's health (3). In women of reproductive age, probiotics have been hypothesized to be beneficial for non-pregnancy related diseases linked to an inflammatory milieu such as Polycystic Ovarian Syndrome (PCOS) (1).

Successful pregnancies require a robust, dynamic and responsive immune system and are primarily dependent on the coordinated balance between the invading trophoblast and receptive maternal decidua (4). Consequently, the decidual immune cells must be optimized to support the development of the fetal-placental unit. These immune cells including macrophages, Natural Killer (NK) cells and T cells, contribute to the establishment of an anti-inflammatory microenvironment and play their respective roles to modulate the adaptive and innate immune system (5). Hence, pregnancy begins in a pro-inflammatory environment that allows for implantation and placentation and subsequently, shifts to an anti-inflammatory stage that facilitates fetal growth and lastly, back to a pro-inflammatory state that ends with labor and delivery (4). The change in states is primarily due to the balance of T helper cells shifting from a pro-inflammatory Th1 profile to a Th2 profile. These responses are also antagonistic to each other which means that a strong Th1 response would tend to have a low Th2 response and vice versa (6). Disruptions in these responses, such as a sudden alteration in cytokine levels and profiles, have the potential to cause spontaneous births or abortions (7).

In recent years, there has been increasing interest in the concept of modulating the gut microbiota to contribute to improving inflammatory states, immunity, and autoimmune diseases. One way to modulate the gut microbiota is through the administration of specific probiotic strains associated with benefits on immunity and overall health. Probiotics are defined by the Food and Agriculture Organization (FAO) of the United Nations as live microorganisms that, when administered in sufficient amounts, can confer a health benefit to the host. Probiotics interact with the Gastrointestinal (GI) mucosa and Gut Associated Lymphoid Tissue (8). These interactions can evoke immune activation signaling or tolerance signaling through the stimulation of cytokine production.

This paper aims to review the evidence about how probiotic administration can help with autoimmune diseases in women of reproductive age, including pregnant and lactating women in relation to changes in cytokine levels and profiles, and the potential effects on the health of women and their children.

### Probiotics and Their Immunomodulatory Properties

Probiotic benefits are strain-specific which means that different strains and dosages elicit different effects on health outcomes, and that there is no universal strain that can address all health outcomes. Among other probiotics, specific *Lactobacillus, Lacticaseibacilus* and *Bifidobacterium* strains have been well-documented in terms of their safety and conferred health benefits. However, modulation of the gut microbiome for health improvement is not always straightforward, as supplementation with one or two probiotic strains is not always associated with detectable changes in microbial composition or activity. Yet, supplementation with probiotics has been suggested to evoke numerous immune benefits through signaling pathways, cytokine and anti-oxidative stress marker expression.

Probiotic bacteria can help induce the secretion of cytokines from intestinal epithelial cells in a strain-specific and dose-dependent manner (9). Ingested probiotics have been reported to interact with enterocytes and dendritic cells, Th1, Th2, and regulatory T cells (Treg) in the intestine. These probiotics must encounter macrophages and dendritic cells to induce the production of anti- or pro-inflammatory cytokines which can evoke subsequent immune signaling pathways. It has been suggested that probiotics can reduce inflammation by stimulating anti-inflammatory cytokines and decreasing pro-inflammatory cytokines which can, in turn, modulate NK cells' activity, inhibit Toll-Like Receptors (TLR) and subsequently, the Nuclear Factor- Kappa B (NF-κB) pathway (10).

**Figure 1:**
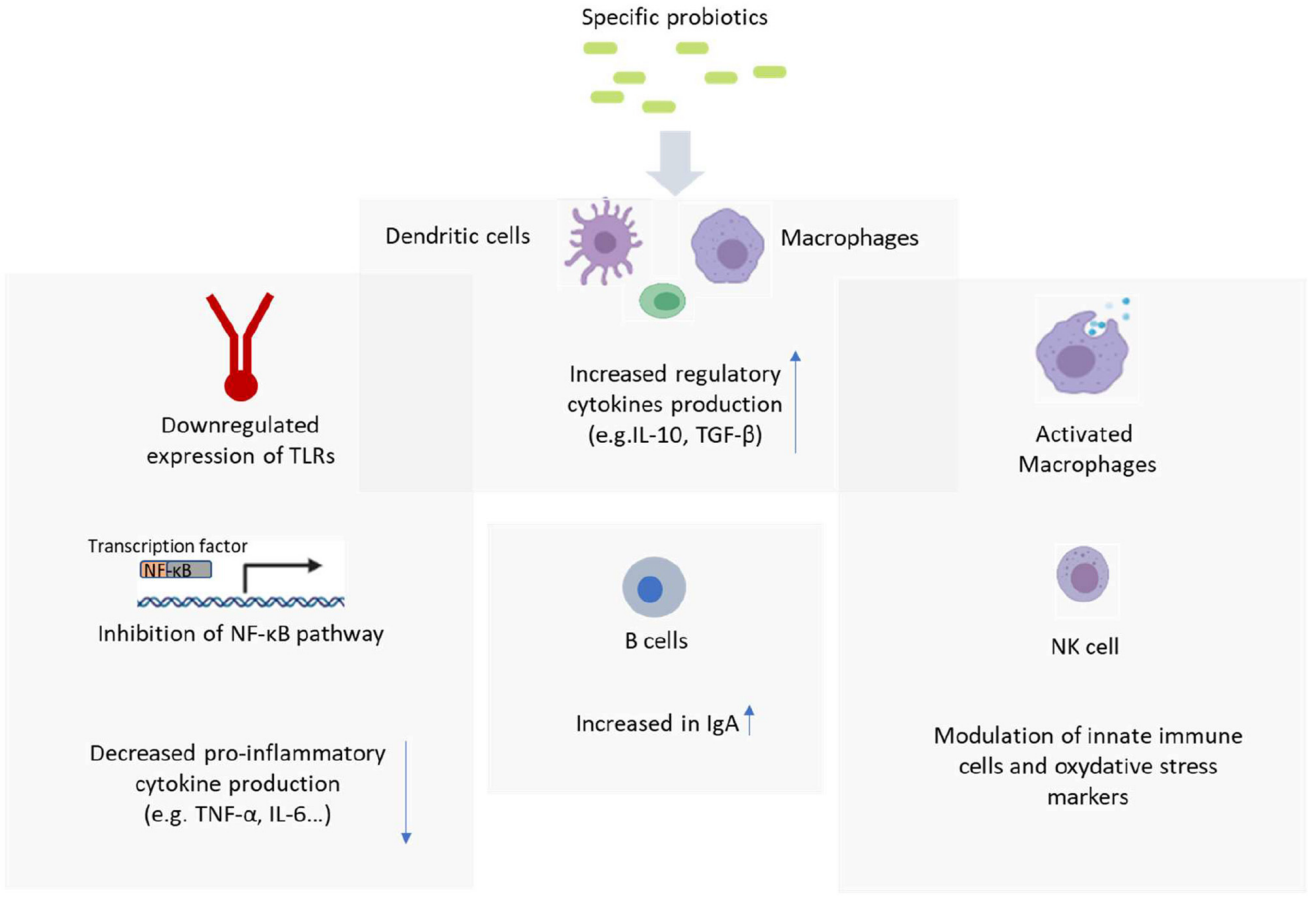
Probiotic mechanism of action. Figure created with BioRender.com.

While the mechanism of probiotics and their effects on the immune system are not entirely understood, this section aims to summarize the suggested mechanisms and their immunomodulatory effects (Figure 1).

#### Inhibition of TLR

Toll-Like Receptors (TLR) are a family of Pattern Recognition Receptors (PRRs) that can recognize a wide range of microbial components. They consist of 11 different proteins (TLR 1—TLR11). The binding of TLR ligands to TLRs can activate the TLR signaling pathway which under certain conditions can exert pro-inflammatory effects (11). TLRs can recognize molecular patterns and activate inflammatory mediators which include the proteins involved in the transcription and signaling of NF-kB and the expression of other TLR ligands (12). TLR ligands are specific in their interaction with TLRs. For example, Lipopolysaccharide (LPS) is specific to TLR 2/4 whereas Flagellin is specific to TLR 5 found on the plasma membrane. TLRs can also be located intracellularly such as TLR 9 in the endosome which is responsive to the DNA as ligands (13). The gut epithelial lining must have a high tolerance to TLR ligands as epithelial cells express minimal TLRs (14), but in states of inflammation and altered gut microbiota, TLRs can be activated and responsive to such ligands to promote further inflammation and oxidative stress and lead to diseases.

Probiotics have been shown to suppress intestinal inflammation *via* the downregulation of TLR expression. Depending on the type of TLR, the reduced expression of TLR can lead to various benefits such as reduced NF-κB activity and other pro-inflammatory expressions (11). Although not yet fully understood, the probiotic(s) used have different interactions in the activity and expression of TLRs. For example, Rautava et al. found that the gene expression of TLRs 1 and 7 was significantly decreased in the placenta whereas TLR3 increased when women received *B. lactis*. However, supplementation with *B.lactis* combined with *L. rhamnosus* GG resulted in a significant decrease only in TLR1 mRNA. There were also carry-over effects to the infants where there was reduced TLR7 mRNA in those whose mothers received *B. lactis* whereas the combination of *B. lactis* and *L. rhamnosus* GG saw a decrease in TLR6 mRNA in the infant intestine (15). Hence, it was concluded that probiotics could develop and strengthen the mucosal immune system managing the inflammatory response. Another study showed an interplay between TLR Single Nucleotide Polymorphisms (SNPs) and the efficacy of *L. rhamnosus* HN001 or *B. animalis* subsp. *lactis* HN019 at reducing the risk of eczema in children compared to placebo (16).

#### Inhibition of the NF-κβ Pathway

NF-κB is a family of transcription factors composed of five members—p65, REL-B, cytoplasmic REL, p50, and p52. Activation of NF-κB involves the phosphorylation and proteolysis of the IκB proteins and the concomitant release and nuclear translocation of the NF-κB factors (17). NF-κB is paramount in controlling the innate and adaptive immune system and is responsive to a wide range of pathogenic stimuli and pro-inflammatory cytokines such as Tumor Necrosis Factor (TNF) and Interleukin-1 (IL-1). These cytokines are highly activated at sites of inflammation in inflammatory bowel disease and metabolic diseases. Activation of NF-κB proteins induces the transcription of more pro-inflammatory cytokines such as IL-6, chemokines, and adhesion molecules which can potentially lead to the onset of inflammatory diseases. IL-6 is a key cytokine of the acute inflammatory response (18). Probiotics can downregulate inflammation by inhibiting various signaling pathways such as the NF-κB pathway which also relates to mitogen-activated protein kinases (MAPK) and PRR pathways (19). Specific strains of probiotics can reduce the binding of NF-κB to the DNA by inhibiting Ikb-βα or ubiquitination phosphorylation and NF-κβ inhibitor degradation and reduce the nuclear translocation of p65. Furthermore, certain probiotics can also inhibit the binding of the LPS to the CD14 receptor, leading to the overall reduction in NF-κβ activity and pro-inflammatory cytokines (20).

#### Modulation of Innate Immune Cells

Natural Killer cells are key cells of the innate immune system and are made up of both NK and NK T cells. NKs can distinguish between healthy and abnormal cells and upon recognition, can elicit the secretion of immune mediators such as cytokine IFN-γ for direct cytolysis of the abnormal cells (21). Together with other phagocytic cells, they prevent infections by forming a barrier against pathogenic microorganisms (22). Being innate, they are non-specific in their recognition of antigens. They also have an innate memory (immune training) and can remember previous pathogenic encounters when attacking again. During pregnancy, decidua NK cells (dNK) dominate the uterine lining and produce a range of angiogenic factors to facilitate vascular stability and function, probably to guarantee arterial remodeling toward the growing fetus (23). These dNKs beyond their role on immune function can also promote a healthy pregnancy and prevent spontaneous abortions. Probiotic strains can increase the cytotoxic potential, proliferation and activation of the NK cells *via* interactions with dendritic cells (24). In a study by Ortiz-Andrellucchi et al., the administration of *L. casei* DN for 6 weeks significantly increased Absolute Variation (AV) of NK cells (CD3^−^CD56^+^) when measured at 10 and 45 days postpartum in peripheral blood treatment (AV: 18.5 cells/μl and −13 cells/μl for *L. casei* and control groups, respectively; *P* = 0.026) (22).

Antigen Presenting Cells (APC) are responsible for the maintenance of immune homeostasis in the GI tract. They are typically made up of Dendritic Cells (DCs), macrophages and other monocytes. APCs produce cytokines and chemokines required for T cell replication, differentiation and response which in turn, initiate antigen-specific immune responses toward potential dangers (25). Such cytokines include IL-12 which causes the differentiation and polarization of Th1 responses and induces cell-mediated immunity such as phagocytosis. Dendritic cells exist throughout the intestine and allow antigen internalization and presentation (25). It has been suggested that probiotics are internalized into APCs to induce maturation which allows them to secrete cytokines for T-cell activation (26). More recently, it has been demonstrated that probiotics (and heat-treated probiotics) could modulate monocyte micro- RNA expression, as a mechanism by which the immunomodulatory cytokine IL-10 was overexpressed through stabilization of the mRNA (27). IL-10 is a regulatory cytokine that has been shown to be modulated by probiotic intervention. Another regulatory cytokine includes TGF-beta. These regulatory cytokines help reduce the initiation or the chronicity of the inflammation by downregulating pro-inflammatory cytokine production.

#### Impact on Oxidative Stress Markers

Oxidative stress can be defined as the imbalance between oxidants and antioxidants in favor of oxidants which leads to subsequent disruptions in redox signaling, modulation and molecular damage. Oxidative stress has been associated with a wide variety of non-communicable diseases and is dependent on oxidation-reduction (redox) homeostasis. Oxidative stress has been shown to play a role in the pathogenesis of autoimmune diseases by downregulating immune tolerance cell number and activity. It is induced by a range of intrinsic factors, foods and environmental factors which generates reactive species such as reactive oxygen species (ROS) and reactive nitrogen species (RNS). Typically, the body can regulate this *via* transcription factors or through reversible protein oxidation reactions, creating a balance between eustress and distress signals. Oxidative stress can be measured using stress markers such as Nitric Oxide (NO), C Reactive Protein (hs-CRP), Glutathione (GSH), Malondialdehyde (MDA), or by measuring the Total Antioxidant Capacity (TAC) which measures the amount of free radicals scavenged.

Nitric Oxide (NO) mediates the relaxation of blood vessels and in mediating oxidative eustress. NO can improve blood pressure, endothelial function and provide other cardiovascular health benefits (28). However, excessive NO production becomes noxious and can induce oxidative damage by forming RNS that can subsequently result in DNA and cellular damage, axonal degeneration and other neurogenerative disorders (29). Probiotics have been suggested to improve endothelial function by modulating the intestinal microbiota and the generation of reactive species and NO bioavailability. This can reduce the risk of cardiovascular diseases and other metabolic dysfunctions (30).

CRP is another marker of systemic inflammation playing a role in innate immunity. They are primarily synthesized in the liver but can also be found in various other cells such as smooth muscle cells, endothelial cells and macrophages. There is growing evidence that CRP plays a role in the inflammatory process through the production of NO and pro-inflammatory cytokines such as IL-6 and TNF-α. Elevated levels of CRP have been found in pathophysiological complications such as insulin resistance, metabolic dysfunctions and chronic inflammatory diseases such as cardiovascular diseases, diabetes and aspects of metabolic syndrome. Probiotics have been suggested to modulate CRP levels *via* an increase in production of Short Chain Fatty Acids (SCFA) in the colon, decreased expression of pro-inflammatory cytokines such as IL-6, and increasing levels of antioxidants and scavengers such as glutathione (GSH) (31, 32).

Glutathione is an antioxidant scavenger that can prevent damage to cellular components such as DNA and lipids by rendering reactive species such as ROS and RNS inactive. They protect such components against free radicals, peroxides, and heavy metals. GSH is known as a master antioxidant and participates in both the antioxidant defense system and metabolic processes (33). Hence, GSH deficiency or imbalance leads to a wide range of pathological and non-communicable diseases such as diabetes. It has been shown that synbiotics and probiotics can increase the synthesis and secretion of GSH through the production of SCFAs (34) and reduced expression of pro-inflammatory cytokines and activity of TLRs (35).

Malondialdehyde (MDA) is a lipid oxidative stress marker and can be formed through lipid peroxidation reactions. Free radicals can oxidize linoleic or arachidonic acid (AA) found to form aldehydes such as MDA. Hence, elevated levels of MDA occur in disease and complications due to oxidative stress (36). The effects of probiotic and synbiotic administration can decrease MDA levels and these changes might be linked to the improved serum lipid profiles. This can include cholesterol-lowering effects, reduced absorption of intestinal cholesterol and modulation of lipid metabolism (37).

## Methods

**Figure 2:**
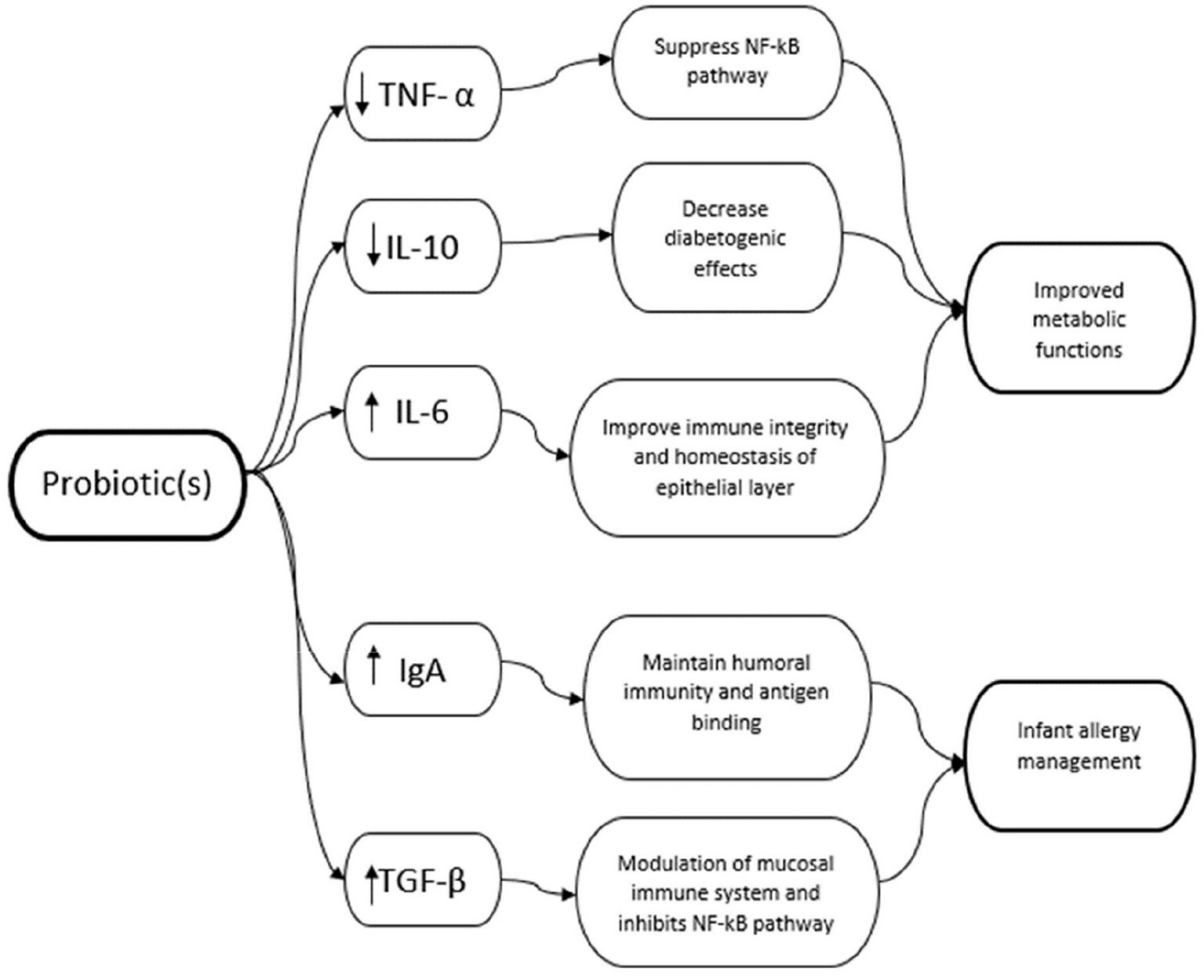
Relevant inflammation markers and health outcomes.

This study aims to investigate the effects of the probiotic intervention on inflammation markers in women and its impact on health outcomes in women and their children. We assessed papers reporting pro-inflammatory and anti-inflammatory cytokines measured in the mother. These indicators were typically measured in the serum and plasma found in the blood and maternal milk during lactation. To narrow the scope of the study, we selected the markers TNF-α, IL-6 but also IgA, and the regulatory cytokines TGF- β, and IL-10 (Figure 2).

All identified English articles published in PubMed on human intervention with probiotics were reviewed. We used a search strategy in accordance with MeSH terminology and used the following search strategy (probiotic* OR synbiotic^*^ OR symbiotic* OR lactobacilli* OR streptococci* OR bifidobacteria* OR saccharum* OR yeast OR yogurt OR bacteria* OR acidophilus OR ferment* OR microorganism*) AND (diet OR supplement OR intake OR consume) AND (preconcept^*^ OR pregnant* OR gestation* OR matern* OR obstetric* OR expectant* OR women) AND (random* OR trial* OR placebo OR blind*). There was a total of 1,931 search results between the search period of 2001 to 2020 where 342 were deemed relevant after reading the abstract. Upon further filtering of the results, 20 publications were focused on probiotic usages and their effects on maternal inflammation markers.

**Figure 3:**
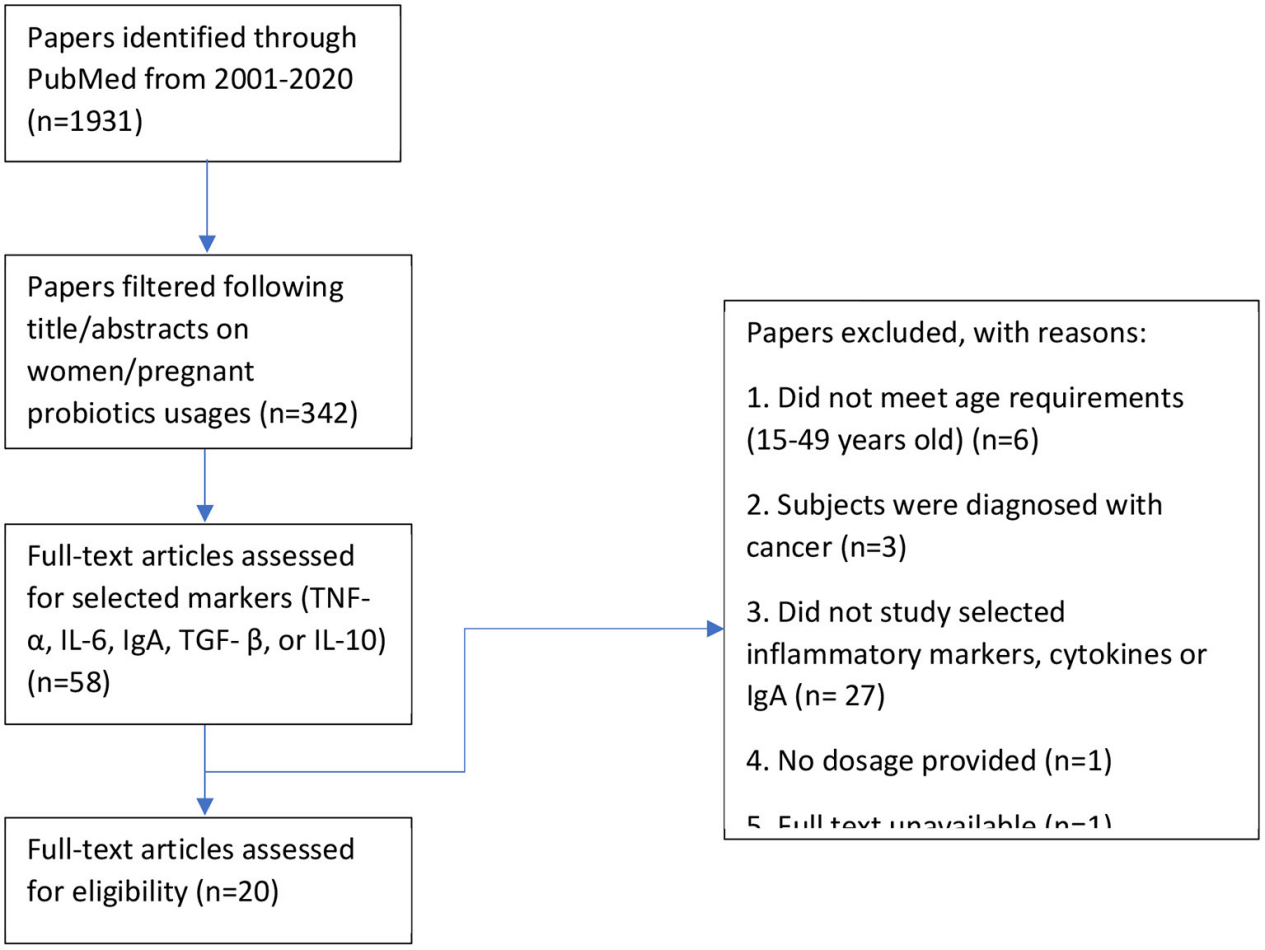
Flow chart of the methodology used to search and select relevant literature results.

This study reviewed all randomized controlled trials studying the effects of probiotics in pregnant women or women of reproductive ages (15–49 years old) that were published in English. All formats of probiotic intakes, such as product supplementation or dietary fortification were included. We excluded articles that included solely prebiotic consumption and women of menopausal ages (>49 years old; Figure 3).

Results on probiotic administration in women of reproductive age or during pregnancy are shown for inflammatory markers (Table 1) and on maternal and infant health and reported across the studies for benefits related to gestational diabetes mellitus, polycystic ovarian syndrome, and infant allergy management.

**Table 1:** Characteristics of the study included in the review.

**References**	**Duration of intervention**	**Number of subjects**	**Population details**	**Probiotic ingredient**	**Dosage**	**TGF-β1**	**TGF-β2**	**IL-10**	**IgA**	**TNF-α**	**IL-6**
Takahashi et al. (38)	2 months from 1 to 3 months postpartum	63	Lactating women at 30–59 days postpartum and has a history of allergy at recruitment	*L. casei,* *B. longum,* *B. coagulans*	1.5 × 10^9^ CFU of *L. casei* LC5, 5 × 10^9^ CFU of *B. longum* BG7, and 2 × 10^8^ CFU of *B. coagulans* 3 tablets a day for about 2 months	**Significant increase in mature breast milk**	**Significant decrease in breast milk**		**Significant decrease in breast milk**		
Baldassarre et al. (39)	4 weeks before the expected delivery date (36th week of pregnancy) until 4 weeks after delivery.	66	Healthy, pregnant women, aged 18–44 years	*S. thermophilus,* *B. breve,* *B. longum,* *B. infantis,* *L. acidophilus,* *L. plantarum,* *L. paracasei,* *L. delbrueckii* *subsp. Bulgaricus*	900 × 10^9^ CFU of the 8 strains	**Significantly higher in mature breast milk**		**Significantly higher in breast milk**	No significant difference		**Significantly higher in colostrum milk**
Prescott et al. (40)	Mothers: 35th week of gestation to 6 months postpartum if breastfeeding Infants: Up to 2 years *via* infant formulation or sprinkled on solid food	105	Pregnant mothers who have or infant's father had a history of being treated for asthma, allergic rhinitis or eczema. Women who were already taking probiotic supplements long term were excluded.	*L. rhamnosus,* *B. animalis* subsp *lactis u*	*L. rhamnosus* at 6 × 10^9^ CFU/day OR *B. animalis* subsp *lactis* strain at 9 × 10^9^ CFU/day	**Significantly higher in both groups in colostrum milk**		No significant difference	No significant difference	No significant difference	No significant difference
Nikniaz et al. (41)	30 days	80	Lactating women who were having a second child who was full term with birth weight between 2,500 and 4,000 g and exclusively breastfed for 3 months	*L. casei,* *L. rhamnosus,* *S. thermophilus,* *B. breve,* *L. acidophilus,* *B. longum,* *L. bulgaricus*	A mixture of 2 × 10^8^ CFU/day, and fructooligosaccharide (394 mg)	No significant difference	**Significant increase in mature milk**		**Significant increase in mature milk**		
Rautava et al. (42)	Mother: 4 weeks before expected delivery Infant: 6 months after birth either through mixing in water	62	Mother-infant with a history of atopic disease	*L. rhamnosus* GG	1 × 10^1^0 CFU/day	No significant difference	**Significantly higher in mature milk**				
Böttcher et al. (43)	From gestational week 36+0 until delivery for mothers First-year of life for infants	109	Families with allergic disease (i.e., one or more family members with eczema, asthma, gastrointestinal allergy, allergic urticaria, or allergic rhinoconjunctivitis)	*L. reuteri*	1 × 10^8^ CFU/day	No significant difference	**Significantly lower in colostrum milk**	**Significantly higher in colostrum milk**	No significant difference	No significant difference	
Boyle et al. (44)	36 weeks gestation to delivery	73	Pregnant women carrying infants at high risk of allergic disease	*L. rhamnosus* GG	1.8 × 10^1^0 CFU/day	No significant difference			**Significantly lower in mature milk**		
Kuitunen et al. (45)	Mothers: 36 weeks of gestation until birth Infant: First 6 months of their life	1,223	Pregnant women carrying a child at a high risk of allergy	*L. rhamnosus* GG *L. rhamnosus* LC705 *B. breve* Bb99 *P. freudenreichii spp. shermanii* JS	*L. rhamnosus* GG, *L. rhamnosus* LC705 and *B. breve* Bb99 1 × 10^1^0 CFU/day of each strain and *P. freudenreichii* ssp *shermanii* JS 4 × 10^9^ CFU/day		No significant difference	**Significant increase in mature milk**	No significant difference		
Huurre et al. (46)	First trimester of pregnancy to end exclusive breastfeeding	140	Prenatal mothers with allergic history	*L. rhamnosus* GG *B. lactis* Bb12	1 × 10^1^0 CFU/day		No significant difference	No significant difference		No significant difference	No significant difference
Hoppu et al. (47)	From early pregnancy to 1 month postpartum	256	Women in early pregnancy from families with a history of allergic disease	*L. rhamnosus* GG *B. lactis* Bb12	10^1^0 CFU/day of each strain			**Significantly higher in both dietary intervention groups compared with control**		**Significantly higher in both dietary intervention groups compared with control**	No significant difference
Ghanei et al. (48)	12 weeks	60	Women between 18 and 45 years old that resided in Tehran and suffered from PCOS	*L. acidophilus* *L. plantarum* *L. fermentum* *L. gasseri*	2 × 10^9^ CFU/day of each strain			**Significant increase**		No significant difference	**Significant decrease**
Meyer et al. (49)	4 weeks	33	Healthy, non-smoking, normocholesterolemic female participants aged between 22 and 29 years	*L. casei* DN114 001	Probiotic yogurt enriched with 3.7 × 10^8^ CFU/ml of *L. casei* DN 114 001 100 g/day for the first 2 weeks then 200 g/day for the next 2 weeks			No significant difference		**Significant increase**	No significant difference
Jafarnejad et al. (50)	8 weeks	82	Women with GDM	*S. thermophilus* *B. breve* *B. longum* *B. infantis* *L. acidophilus* *L. plantarum* *L. paracasei* *L. delbrueckii* subsp. *bulgaricus*	225 × 10^9^ CFU/capsule of eight strains of lactic acid bacteria			No significant difference		**Significant decrease**	**Significant decrease**
Yang et al. (51)	12 weeks	86	Asymptomatic pregnant (before 17 weeks of gestation) women who had an Intermediate or Bacterial Vaginosis Nugent score at 13 weeks.	*L. rhamnosus* GR-1 *L. reuteri* RC-14	5.0 × 10^9^ CFU/day each			No significant difference		No significant difference	No significant difference
Singh et al. (52)	4 weeks	22	Healthy, non-pregnant women	*S. thermophilus* *B. breve* *B. longum* *B. infantis* *L. acidophilus* *L. plantarum* *L. paracasei* *L. delbrueckii* subsp. *Bulgaricus*	225 × 10^9^ CFU of a mixture of eight strains			No significant difference		No significant difference	No significant difference
Vitali et al. (53)	4 weeks (week 33 to 37 of gestation)	27	Healthy women during late pregnancy	*S. thermophilus* *B. breve* *B. longum* *B. infantis* *L. acidophilus* *L. plantarum* *L. paracasei* *L. delbrueckii subsp. Bulgaricus*	9 × 10^1^1 CFU/day			No significant difference		No significant difference	No significant difference
Lorea Baroja et al. (54)	30 days	40	20 subjects with IBD and 20 healthy controls with no known or suspected intestinal abnormalities. 15 of the IBD patients had Crohn's disease	*L. rhamnosus* GR-1 *L. reuteri* RC-14	1 × 10^3^ CFU/ml of *L. reuteri* RC-14 and 2 × 10^7^ CFU/ml of *L. rhamnosus* GR-1. 125 g of yogurt consumed a day			No significant difference in IBD patients.		No significant difference in IBD patients	
Kabeerdoss et al. (55)	3 weeks	26	Healthy women aged 18–21	*B. lactis* Bb12	10^9^ CFU/day				**Significantly higher**		
Hajifaraji et al. (56)	8 weeks	56	Between 24 and 28 weeks and 6 days gestation, diagnosed with GDM	*L. acidophilus* La5 *B. lactis* Bb12 *S. thermophilus* *L. delbrueckii bulgaricus*	Sum of at least 4 × 10^9^ CFU/day					**Significantly decreased**	No significant difference
Asemi et al. (57)	9 weeks in the 3rd trimester of pregnancy	70	Pregnant women, primigravida, aged 18–30 years old who were carrying singleton pregnancy at their third trimester	*L. acidophilus* La5 *B. lactis* Bb12	Probiotic yogurt enriched with a total of 200 × 10^7^ CFU/day					No significant difference	

*Significant differences in cytokines or IgA are highlighted in bold*.

## Probiotic Administration In Women Of Reproductive Age

### Clinical Evidence on Inflammatory Markers

Meyer et al. administered a probiotic yogurt enriched with *L. casei* DN 114 001 at 3.7 × 10^8^/ml and a conventional yogurt on healthy women between 22 and 29 years old for 4 weeks (*n* = 33) (49). Participants were asked to consume 100 g of yogurt for the first 2 weeks then 200 g of yogurt for the next 2 weeks. Here, there was a significant increase in TNF-α levels during the first 2 weeks of intake in the conventional and probiotic yogurt group and remained higher than baseline in the next 2 weeks. IL-10 showed a significant decrease after the probiotic yogurt consumption during the first 2 weeks but was not significantly different from the conventional yogurt group at the final evaluation. IL-6 showed no significant difference during the study period. Results concluded that both conventional and probiotic enriched yogurt enhanced the stimulated production of pro-inflammatory cytokines by myeloid cells such as macrophages and NK cells. Asemi et al. administered a probiotic yogurt enriched with *L. acidophilus* La5 and *B. lactis* Bb12 a total of 200 × 10^7^ CFU/day on pregnant women (*n* = 70) for 9 weeks during the 3rd trimester of pregnancy (57). Results showed that the probiotic yogurt had no significant effects on serum TNF-α. Here results are thus conflicting, and more study are needed to reach a conclusion.

Despite probiotic yogurts exhibiting minimal changes in immunomodulatory markers, it still appears useful in terms of immune function. A study by Meyer et al. has shown that higher cytolytic activity of NK cells has been exhibited after yogurt consumption which can explain better defense against pathogenic microorganisms, protection against infections and acute inflammation (58).

Lastly, studies showed that oral probiotic administration may be ineffective in modulating vaginal cytokine profiles. Yang et al. administered *L. rhamnosus* GR-1 and *L. reuteri* RC-14 at 5.0 × 10^9^ CFU each for 12 weeks on asymptomatic pregnant women (before 17 weeks of gestation) who had an Intermediate or Bacterial Vaginosis Nugent score at 13 weeks (*n* = 86) (51). Results showed that there were no significant differences in levels of IL-10, TNF-α, and IL-6 at the end of the intervention in vaginal samples. It was hypothesized that this could have been due to insufficient dosages of probiotics to displace the indigenous *lactobacilli*, resulting in minimal changes in microbiota function and potentially no changes in the inflammation markers. A study in Italy conducted for 4 weeks from week 33–37 weeks of gestation also administered VSL#3 at 9 × 10^11^ CFU/capsule per day on healthy women (*n* = 27) and saw no significant differences in TNF-α, IL-6, and IL-10 at the end of the intervention in vaginal samples (53). One reason suggested was that VSL#3 could have contained too many species of organisms and in turn, canceled the stimulatory effect of other constituents (59). Another study by Singh et al. also administered VSL#3 at 112.5 × 10^9^ CFU/capsule twice a day in healthy non-pregnant women (*n* = 22) for 4 weeks results showed that there were no significant differences in levels of TNF-α, IL-6, and IL-10 at the end of the intervention (52).

Results in health and disease for the modulation of inflammatory markers are still highly conflicting. Modulation of TNF-α and IL-6 or the regulatory cytokine IL-10 may depend on the specificity of the probiotics, the health or disease state and the timing of the intervention. Further studies are thus needed to confirm the relevance of these markers as a proxy for probiotic clinical response in human clinical trials.

### Polycystic Ovarian Syndrome and Gestational Diabetes Mellitus

PCOS affects both reproductive and metabolic functions, causing infertility, obesity and insulin resistance in women. Similar to metabolic syndrome, it is characterized by a constellation of symptoms which consist of hyperandrogenism, ovarian dysfunction, and polycystic ovarian morphology (60). It is also associated with metabolic disorders such as insulin resistance (61), type 2 diabetes, and other cardiovascular diseases (62). PCOS is caused by the interaction between numerous genes which affect neuroendocrine and metabolic functions, inducing metabolic endotoxemia and circulation of LPS, causing low-grade chronic inflammation, cardiometabolic changes, increased NF-KB activation, and oxidative stress (63). PCOS has been shown to chronically activate the immune system, regardless of obesity and recent studies have suggested that the gut microbiota can be a potential pathogenic factor in the development of PCOS.

A randomized, double-blinded and placebo-controlled conducted in Iran in 2018 administered *L. acidophilus, L. plantarum, L. fermentum*, and *L. gasseri* at 2 × 10^9^ CFU of each strain per day for 12 weeks to women who suffered from PCOS (*n* = 60) (48). Results showed that serum IL-10 significantly increased in the group with probiotic supplementation. However, serum TNF-α showed no significant differences and IL-6 showed a significant decrease in both groups. IL-10 showed a significant increase in the probiotics group as compared to the placebo group. Another trial with the same combination of probiotics at 2 × 10^9^ CFU per strain, co-administered with 200 μg/day selenium as selenium yeast, showed significant reductions in weight, serum insulin levels and homeostatic model of assessment for insulin resistance and a significant increase in glucose and insulin (64). It also significantly decreased serum triglycerides and LDL levels. While more research is needed, evidence may suggest that these 4 lactobacilli probiotic strains can be useful in modulating inflammation markers and help with metabolic dysfunctions but other studies need to confirm these findings.

Similar to other chronic diseases, it is unclear whether the gut microbiota dysbiosis causes PCOS or the other way round (65). However, the intestinal microbiota plays a role in PCOS pathogenesis reason why, probiotic usage could be a nutritional intervention to help prevent or manage PCOS. Evidence has shown to be useful in managing the metabolic profiles in women with PCOS. A meta-analysis in 2020 concluded that synbiotics and probiotics can improve hormonal and inflammatory indices in this population (66). Such inflammatory indices typically contribute to impaired glucose tolerance, insulin sensitivity and other complications. Synbiotic and probiotic administration can modulate glucotoxicity and manage the pathophysiological complications that come with PCOS.

GDM is defined as any form of glucose intolerance during pregnancy. During pregnancy, metabolic changes occur that cause adipose tissue accretion and the gradual development of insulin resistance to facilitate fetal nutrition (67). It has become a rising concern as GDM is a risk factor for developing other complications such as hypertension, preeclampsia, type 2 diabetes, and other cardiometabolic diseases. High glucose levels stimulate pro-inflammatory effects by inducing TLR activation by causing an increase in TLR2 and TLR4 expression, which ultimately results in the NF-kB activation and further pro-inflammatory cytokine secretion (12). The increased TLR4 expression also activates NF-κB and mitogen-activated protein kinase (MAPK) signaling pathway, leading to the increased transcription of genes involved in inflammation and this results in the feed-forward loop of inflammation levels. This elevated level of inflammation can result in insulin secretion impairment and insulin resistance, leading to metabolic inefficiencies and ultimately, the development of GDM. GDM results in elevated glucose levels and often remains unregulated. Hence, it has recently been of interest to investigate strategies to bring down these elevated levels of inflammation to help with GDM risk and management. Probiotics can regulate the secretion of pro-inflammatory mediators bringing down the risk of insulin resistance and thus the onset of GDM (68).

TNF-α is a pro-inflammatory cytokine that is a major regulator of the NF-kB and MAPK pathways. These cytokines are capable of promoting the proliferation of several cells and signals apoptosis, inevitably playing a part in immunity (69). Interleukin-6 (IL-6) is another pro-inflammatory cytokine that mediates interactions between the immune system and the Central Nervous System (CNS) and is disruptive to its functions (70). It has been suggested to be associated with GDM due to its diabetogenic actions and can interfere with normal insulin resistance (71). Lastly, IL-10 is regarded as an anti-inflammatory cytokine that is essential in maintaining the immune integrity and homeostasis of tissue epithelial layers. Other suppressive cytokines that inhibit pro-inflammatory responses such as lesion production for cardiovascular health. These cytokines are produced by a wide variety of cells including leukocytes, macrophages, dendritic cells, NK cells and T cells to help control acute and chronic inflammation either by regulating other immune responses such as T cell response or by modulating secretion and expression of pro-inflammatory cytokines (72).

As the development of PCOS and GDM involves a multitude of factors such as insulin resistance and chronic low-grade inflammation, one can hypothesize that pro-inflammatory cytokines play a role in the onset of metabolic diseases (73). Several studies have shown that probiotics can regulate TNF-α, IL-6, and IL-10 which are associated with metabolic dysfunctions.

Administration of *L. acidophilus* La5, *B. lactis* Bb12, *S. thermophilus* STY-31, and *L. delbrueckii* bulgaricus LBY-27 at a sum of at least 4 × 10^9^ CFU/day in women diagnosed with GDM, for 8 weeks during pregnancy (*n* = 64) (56) showed significantly lower serum TNF-α in the probiotic group as compared to the placebo. However, there were no significant differences between the 2 groups in serum IL-6 levels. Another study in 2016 administered VSL#3 containing 112.5 × 10^9^ CFU/capsule of eight strains; *S. thermophilus, B. breve, B. longum, B. infantis, L. acidophilus, L. plantarum, L. paracasei*, and *L. delbrueckii* subsp. *bulgaricus* (50). The administration of VSL#3 was conducted in women with GDM for 8 weeks (*n* = 82). Here, the probiotic administration resulted in a significant decrease in levels of TNF-α (−0.62 ± 1.0 vs. 0.45 ± 0.8 pg/mL; *p* = 0.04) and IL-6 (−0.44 ± 0.5 vs. 0.33 ± 0.42 pg/mL; *p* = 0.04) as compared to the placebo. However, IL-10 showed no significant difference between the probiotic group and the placebo group (0.74 ± 4.4 vs. −0.4 ± 5.1; *p* = 0.54).

Despite observed inconsistencies in changes in the cytokine profiles after probiotic administration, the evidence suggests that probiotic administration is safe and as far as efficacy, it is either neutral or beneficial. Furthermore, it is probably not just the changes in cytokine levels that contribute to these beneficial changes. A meta-analysis in 2020 showed that the reduction of fasting glucose in pregnant women without GDM using a range of probiotics is very small and by itself may not be clinically relevant. Furthermore, probiotic administration did not reduce the incidence of GDM (65). However, it has been shown that probiotics can modulate gestational glucose haemostasis and reduce glucotoxicity by increasing insulin sensitivity (74). Hence, probiotics as a unique approach may not be a meaningful strategy in the prevention of GDM but could be considered as part of more holistic approaches to improve glycemic response and glucose metabolism in women. We hypothesize that probiotics may be useful to reduce the risk of GDM when used in conjunction with other strategies such as dietary interventions, obesity management or when used in combination with other bioactives. Lastly, a recent meta-analysis showed that probiotic intervention in GDM women can improve maternal HDL-cholesterol and markers of inflammation and oxidative stress, and can reduce the incidence of hyperbilirubinemia in the newborn (75). Hence, probiotic administration may be beneficial in pregnant women with GDM to manage GDM complications and improve some infant outcomes, however further research is needed.

While not many studies have investigated probiotics for GDM or PCOS prevention, probiotic/synbiotic administration seems to benefit glycemic profiles, thus reducing a risk factor for metabolic complications and potentially related diseases such as GDM and PCOS while proving safe for consumption even during pregnancy. As the onset of GDM or PCOS typically includes a multitude of factors, managing one risk factor through probiotic administration may still be beneficial.

### Infant Allergy Risk Reduction

Breast milk composition can help with infant intestinal inflammatory conditions and the reduction of risk of allergic diseases (76). Secretory Immunoglobulin A (IgA) can help maintain humoral immunity by binding to antigens, thereby limiting their access to the epithelium (77). It has been shown that infants are unable to produce their protective levels of IgA until almost 30 days after birth and hence, are reliant on other factors such as maternal breastfeeding to attain adequate levels of IgA (78). Probiotics have been shown to stimulate the production of secretory IgA (19) in human milk or the intestinal tract of infants. Hence, the stimulated production and higher levels of IgA antibodies in human milk may have transferred over to the infants during breastfeeding and can help with antigen-epithelium related diseases such as allergy management.

Other inflammatory markers often discussed in human milk are also transforming growth factor-beta (TGF-β) and IL-10. TGF-β suppresses inflammatory responses by inducing Tregs and promoting B-cell IgA production (79). Rigotti et al. demonstrated allergic mothers have a reduced TGF-β1 in breast milk and colostrum that may affect the modulation of the mucosal immune system and facilitate the development of allergy (80). IL-10 negatively regulates cell-mediated immunity directly or *via* suppression of antigen-presenting cell functions including IL-12 production (9) and can also inhibit NF-κB (81). It has also been suggested that IL-10 can suppress IL-12 and consequently IFN-γ production which leads to a Th2 or T regulatory response (82). Niers et al. suggested that the prevention of atopic eczema by probiotics could be through TLRs which have a key role to play in inhibiting the NF-κB and maintaining mucosal and intestinal homeostasis (83).

Prescott et al. suggested that the prenatal/postnatal administration of *L. rhamnosus* HN001 can reduce the rate of eczema by increasing the level of cord blood interferon-γ and consequently, change the composition of intestinal flora in children, thus modulating the immune system and allergy risk. A study conducted in New Zealand in 2008 administered *L. rhamnosus* HN001 at 6 × 10^9^ CFU/day or *B. animalis* subsp *lactis* HN019 at 9 × 10^9^ CFU/day from 35th week of gestation up to 6 months postpartum and subsequently, up to 2 years in the infant *via* formula or solid food. Breast milk samples were analyzed at 1 week, 3 months, and 6 months of lactation. Results showed that the probiotic administration resulted in significantly higher levels of TGF-β1 (*p* = 0.028) in breast milk (week 1) as compared to the placebo group. Effects of this were more pronounced in the *B. lactis* HN019 group (*P* = 0.041) with a similar trend in the *L. rhamnosus* HN001 group (*P* = 0.075). However, such differences were no longer observed in mature breast milk (3 and 6 months). Furthermore, there were no significant differences observed in breast milk TNF-α, IL-6, IL-10, and IgA throughout the administration period (40). Interestingly, the *L. rhamnosus* group supplementation group showed a significantly reduced risk of eczema at 2 years (HR 0.51; 95% CI, 0.30–0.85) compared to the placebo which was not observed in the *B. lactis* group (HR 0.90; 95% CI, 0.58–1.41) (74) despite showing similar changes in breast milk cytokine levels. It may suggest there may be a multitude of other factors linked to *L. rhamnosus* immunoregulation of infant allergy that *B. lactis* HN019 does not have and confirm that infant atopic dermatitis risk reduction is not solely reliant on breast milk cytokine profiles.

Another study found that *L. rhamnosus* may be ineffective in affecting breast milk anti-inflammatory markers such as TGF-β levels. A study administered *L. rhamnosus* GG at 10^10^ on mothers with a history of atopic disease for 4 weeks before expected delivery (42). Results showed that mature breast milk (3 months) TGF-β1 showed no significant differences compared with the placebo group. However, TGF-β2 in the breast milk of mothers receiving the probiotics was higher than that in the breast milk of mothers receiving placebo. Probiotic administration was still effective in the prevention of early atopic diseases in children at high risk (84). Even at higher dosages of *L. rhamnosus* GG, this probiotic strain may prove ineffective in modulating breast milk inflammatory markers. One study administered *L. rhamnosus* GG at 1.8 × 10^10^ CFU/day on pregnant women with a high allergic disease risk from 36 weeks of gestation to delivery (*n* = 73) (44) and found a lower level of total IgA seen in the 28-day mature milk as compared to the placebo group but no significant differences were seen in total IgA in the 7-day breast milk samples and no significant difference in breast milk TGF-β1 compared with the placebo group at 7 or 28 days.

Furthermore, results vary when *L. rhamnosus* GG is consumed in conjunction with other probiotic strains. A similar finding by Hurree et al. also administered *L. rhamnosus* GG and *B. lactis* Bb12 at 10^10^ CFU/day in pregnant women with an allergic history from the first trimester of pregnancy to the end of exclusive breastfeeding (*n* = 140) and found no significant differences in TGF-β1, TNF-α, IL-10, and IL-6 on the colostrum or mature (1 month) breast milk samples compared with the placebo group (46).

Kuitunen et al. administered *L. rhamnosus* GG (ATCC 53103) 10^10^ CFU, *L. rhamnosus* LC705 10^10^ CFU, *B. breve* Bb99 10^10^ CFU and *P. freudenreichii* ssp *shermanii* JS 4 × 10^9^ CFU in mothers from 36 weeks of gestation until birth and to infants during the first 6 months of life (with 0.8 g of prebiotics with infants; *n* = 1,223) (45). Colostrum milk and mature breast milk (3 months) were analyzed. Results showed that probiotic intervention showed no significant differences compared to the placebo group in colostrum TGF-β2 levels and mature breast milk TGF-β2. However, the probiotic intervention increased IL-10 levels in the mature breast milk but did not influence their levels in colostrum. Lastly, probiotic intervention showed no differences as compared to the placebo group in total IgA in colostrum milk and mature milk. An interesting finding was that high TGF-β2 in mature BM was associated with more allergic disease and eczema at 2 years and was independent of probiotic treatment. As mentioned above, it appears that immunological development of infant allergy and the protective effect of probiotics are not necessarily mediated by immunomodulatory factors in breast milk but may be attributed to other components.

Apart from the administration of *L. rhamnosus* GG, other probiotic combinations have been used to investigate its effects on breast milk inflammatory markers. Baldassarre et al. administered Vivomixx^®^ in healthy pregnant women from 4 weeks before the expected delivery date until 4 weeks after delivery (*n* = 66) (39). Vivomixx^®^ contained 8 strains at a sum of 900 × 10^9^ CFU of *S. thermophilus* DSM 24731, *B. breve* DSM 24732, *B. longum* DSM 24736, *B. infantis* DSM 24737, *L. acidophilus* DSM 24735, *L. plantarum* DSM 24730, *L. paracasei* DSM 24733, and *L. delbrueckii* subsp. *bulgaricus* DSM 24734. Results showed that breast milk TGF-β1, IL-6, and IL-10 were significantly higher in colostrum and mature (30 days) breast milk of the probiotic group compared to the control group but there were no significant differences observed for levels of IgA. One interesting observation was that IgA was significantly higher in newborn stools in the probiotic group compared to the control group. This may suggest that the increase in TGF-β1 in the breast milk of the probiotic group could result in higher IgA levels but is instead found in amniotic fluid as opposed to breast milk to create a more effective mucosal immune system. Hence, this shows probiotic supplementation has the potential to confer its health benefits to the infant despite certain breast milk inflammation markers showing no significant differences.

Another study conducted in Iran in 2013 administered a synbiotic combination of *L. casei* PXN 37, *L*. PXN 54, *S. thermophilus* PXN 66, *B. breve* PXN 25, *L. acidophilus* PXN 35, *B. longum* PXN 30, *L. bulgaricus* PXN 39 at a mixture of 2 × 10^8^ CFU, and fructo-oligosaccharide (394 mg) daily for 30 days in lactating women who breastfed exclusively for 3 months (41). Results showed that there was an increase in TGF-β2 levels of breast milk and IgA while no significant differences were seen in the placebo group. However, no significant differences were observed in breast milk TGF-β1 levels. An open-label pilot trial administered 5 × 10^9^ CFU of *L. casei* LC5, 5 × 10^9^ CFU of *B. longum* BG7, and 2 × 10^8^ CFU of *B. coagulans* SANK70258 per day for 2 months from 1 to 3 months postpartum (85). Results showed that there were no significant differences in median TGF-β1 and TGF-β2 from the 1 to 3 months postpartum. IgA levels decreased in both the probiotic and the placebo groups. One notable difference was that the median TGF-β1 decreased from 1 to 2 months postpartum followed by an increase from 2 to 3 months. To the best of our knowledge, the studies by Nikniaz et al. (41) and Takahashi et al. (38) were the only two studies that focused on probiotic administration solely during the lactation phase These 2 studies further highlight that probiotics act in a strain-specific manner and the discrepancy in results was likely due to the different probiotic strains, dosages and intervention formats used.

Lastly, few studies showed a decrease in anti-inflammatory markers. One study in Sweden found that *L. reuteri* ATCC 55730 at 1 × 10^8^ CFU/day was supplemented through coconut and peanut oil drops. Supplementation was done from 36 weeks of gestation until delivery and inflammation markers were measured form colostrum and mature milk (1 month) (*n* = 109) (43). Results showed that there was a significantly lower amount of TGF- β2 and a significantly higher amount of IL-10 in colostrum milk as compared to placebo. However, there were no significant differences found in mature milk. Furthermore, there were no significant differences in levels of TGF- β1, IgA, and TNF-α in either colostrum or mature milk.

Research has remained inconsistent on how probiotic administration can affect the cytokine profile in breast milk, the fetus and their translated effects on infant outcomes. Ultimately, this depends on the probiotic strain, dosages, and period of intervention used. Despite similar changes in breast milk cytokine profile using two different probiotic strains, as demonstrated by Wickens et al. (86), there were not always associated with an allergy risk reduction in infants. Even if there were differences in breast milk cytokine profile in some studies, it does not seems to associate with infant outcomes if we compare Wickens et al. (86) and Kalliomäki et al. (84). Hence, it may be more meaningful to understand the strain specificity of probiotics and how it impacts allergy risk reduction than use cytokine as surrogate marker management in terms of immunological pathways. Even if infant allergy prevention is associated with the breast milk inflammatory markers, it appears to not always be associated with TGF-β, IL-10, or IgA and could perhaps be related more closely to fatty acids and/or human milk oligosaccharides that were not discussed (86).

However, one strategy that could be an administration started during pregnancy and be continued during lactation if possible. Despite the differences in breast milk immunomodulatory factors, one consistent result from these studies is that administration should start during late pregnancy, continued postnatally and should be fed to the infant even after breastfeeding for allergy management. We also recommend a probiotic species of *L. rhamnosus* HN001 *or L. rhamnosus* GG to be used as results may show its better competency in being an immunomodulatory agent as compared to other species and better effects on infant allergy risk management. Furthermore, a systematic review and meta-analysis conducted by Garcia-Larsen et al. concluded that probiotics supplements such as *L. rhamnosus* administered at a dosage between 1 and 10 × 10^9^ CFU per day taken from 36 to 38 weeks gestation and through the first 3 to 6 months of lactation may reduce the risk of eczema in children. One precautionary measure is to avoid administration during early pregnancy as it has been associated with adverse effects such as intestinal disorders or immune deficiency (85).

## Conclusion, Limitations, And Future Directions

We cannot strongly conclude on the impact of probiotic interventions on markers of inflammation and health outcomes in women of reproductive age and their children, due to the heterogeneity of the study results. Further studies should be done to focus on the contribution of the immunomodulatory markers and their association with health impacts on the subject. Our focus was maternal inflammation markers, and we did not explore the carry-over effects to the infant inflammatory markers due to the absence of data on infant inflammatory markers in cord blood.

Lastly, we recognize that inflammation markers may also be representative of a current symptomatic or asymptomatic infection and not always linked to the observed clinical outcome of diseases. These and other confounding factors such as lifestyle modifications, age, diet and the environment can contribute heavily to the observed changes, and/or offset the effects of probiotic administration. The assessment of probiotic efficacy in well-designed randomized placebo control trials is thus essential.

Observation made in this paper should be taken with caution and should not be generalized to all populations or all probiotics. The study designs mentioned in the paper are inherently different, making it difficult to determine if the evidence presents a global representation of the effects of probiotic administration. Current observations tend to conclude toward probiotic strain-specific effects.

Research remains inconsistent on the changes in immunomodulatory properties through probiotic administration. However, probiotic administration has been shown to evoke beneficial or neutral effects on non-communicable diseases and some of their risk factors. Beyond non-communicable diseases and cytokine profiles, specific probiotic strains appear to reduce the risk or duration of infections, but this was not discussed in this paper. In conclusion, specific probiotic strain or specific probiotic mix administration may provide beneficial effects in premenopausal women. However, proper assessment of probiotic strains, dosages, and intervention duration is desirable to allow a good interpretation of the results of future intervention studies. Future studies need to further evaluate the mechanisms of probiotics and their impact on inflammatory markers.

## Author Contributions

ST, IS-Z, and LF initiated the project. KK and CB gathered the data and prepared the publication. KK, CB, LF, IS-Z, AI, and ST drafted, reviewed, and accepted the last version of the documents. All authors contributed to the article and approved the submitted version.

## Conflict of Interest

KK, LF, IS-Z, ST, AI, and CB were employed by Nestlé Research during the conduct of the study.

## Publisher's Note

All claims expressed in this article are solely those of the authors and do not necessarily represent those of their affiliated organizations, or those of the publisher, the editors and the reviewers. Any product that may be evaluated in this article, or claim that may be made by its manufacturer, is not guaranteed or endorsed by the publisher.

## Abbreviations

CFU: Colony Forming Unit
CRP: C-Reactive Protein
GDM: Gestational Diabetes Mellitus
GSH: Glutathione
HDL: High-Density Lipoprotein
IgA: Immunoglobulin A
IL: Interleukine
NK: Natural Killer
TLR: Toll-Like Receptors
GSH: Glutathione
LPS: Lipopolysaccharide
MAPK: mitogen-activated protein kinases
NF-κB: Nuclear Factor-Kappa B
PCOS: Polycystic Ovarian Syndrome
PRRs: Pattern Recognition Receptors
ROS: Reactive Oxygen Species
SCFA: Short Chain Fatty Acids
SNPs: Single Nucleotide Polymorphisms
TGF: Transforming Growth Factor
TNF: Tumor Necrosis Factor.
